# Evaluation of Activity and Emergence of Resistance of Polymyxin B and ZTI-01 (Fosfomycin for Injection) against KPC-Producing Klebsiella pneumoniae

**DOI:** 10.1128/AAC.01815-17

**Published:** 2018-01-25

**Authors:** John K. Diep, Rajnikant Sharma, Evelyn J. Ellis-Grosse, Cely S. Abboud, Gauri G. Rao

**Affiliations:** a UNC Eshelman School of Pharmacy, University of North Carolina, Chapel Hill, North Carolina, USA; b University at Buffalo, State University of New York, Buffalo, New York, USA; c Zavante Therapeutics, Inc., San Diego, California, USA; d Instituto Dante Pazzanese de Cardiologia, São Paulo, Brazil

**Keywords:** polymyxin B, fosfomycin, extended dosing, carbapenemase, Klebsiella pneumoniae, pharmacodynamics, resistance

## Abstract

ZTI-01 (fosfomycin for injection) is a broad-spectrum antibiotic with a novel mechanism of action and is currently under development in the United States for treatment of complicated urinary tract infections. Globally, fosfomycin and polymyxin B are increasingly being used to treat multidrug-resistant Gram-negative infections. The objectives were to evaluate the pharmacodynamic activity of polymyxin B and fosfomycin alone and in combination against KPC-producing Klebsiella pneumoniae and to assess the rate and extent of emergence of resistance to different antibiotic regimens. Two clinical isolates, BRKP26 (MIC of polymyxin B[MIC_PMB_], 0.5 mg/liter; MIC of fosfomycin [MIC_FOF_], 32 mg/liter) and BRKP67 (MIC_PMB_, 8 mg/liter; MIC_FOF_, 32 mg/liter) at an initial inoculum of 10^7^ CFU/ml, were evaluated over 168 h in a hollow-fiber infection model simulating clinically relevant polymyxin B (2.5-mg/kg loading dose as a 2 h-infusion followed by 1.5-mg/kg dose every 12 h [q12h] as a 1-h infusion) and fosfomycin (6 g q6h as a 1-h or 3-h infusion) regimens alone and in combination. Population analysis profiles (PAPs) and MIC testing were performed to assess emergence of resistance. Polymyxin B or fosfomycin monotherapy was ineffective and selected for resistance by 24 h. Polymyxin B plus a fosfomycin 1-h infusion demonstrated sustained bactericidal activity by 4 h, with undetectable colony counts beyond 144 h. Polymyxin B plus a fosfomycin 3-h infusion demonstrated bactericidal activity at 4 h, followed by regrowth similar to that of the control by 144 h. PAPs revealed resistant subpopulations by 120 h. The combination of polymyxin B and a fosfomycin 1-h infusion is a promising treatment option for KPC-producing K. pneumoniae and suppresses the emergence of resistance. Further evaluation of novel dosing strategies is warranted to optimize therapy.

Carbapenem-resistant Enterobacteriaceae are categorized as an urgent threat by the U.S. Centers for Disease Control and Prevention (1). More specifically, carbapenemase-producing Enterobacteriaceae (CPE) pose a major public health concern due to their increasing global prevalence (2, 3). Klebsiella pneumoniae carbapenemase (KPC) enzymes confer broad-spectrum resistance to β-lactam agents including carbapenems (4–6). Due to resistance to nearly all currently available antibiotics, CPE infections are often difficult to treat and are an independent predictor of mortality, with high mortality rates of between 26% and 44% (7, 8).

Limited therapeutic options have led to the revival of older antibiotic agents such as the polymyxins and fosfomycin (9, 10). Treatment with agents like polymyxin B or colistin (polymyxin E) is becoming more common due to their excellent *in vitro* activity against CPE (11, 12). However, reports of polymyxin resistance are increasing, and more resistance has been seen in regions with high polymyxin use (12, 13). A large outbreak of KPC-producing K. pneumoniae causing bloodstream infections was reported in a hospital in Italy (14), and the proportion of polymyxin-resistant cases increased from 12% in 2011 to 65% in 2012. KPC-producing K. pneumoniae bacteria resistant to polymyxins have been reported globally (15–18) and threaten the utility of these last-line antibiotics.

Fosfomycin is another “old” antibiotic that shows *in vitro* activity against CPE (19–21). Its unique mechanism of action involves the inhibition of UDP-*N*-acetylglucosamine-3-enolpyruvyl transferase (MurA), an enzyme that catalyzes the first step in peptidoglycan synthesis (22). Oral fosfomycin has long been used for urinary tract infections due to the high concentrations achievable in urine, but the limited bioavailability and significant gastrointestinal side effects observed after multiple doses limit its use for deep-seated infections. Intravenous fosfomycin (known as ZTI-01) is under development in the United States for complicated urinary tract infections. Given fosfomycin's broad-spectrum activity, the ability to achieve significant concentrations, and the variety of approved indications outside the United States (including hospital-acquired bacterial pneumonia/ventilator-associated bacterial pneumonia and meningitis), fosfomycin may be considered well suited for the treatment of multidrug-resistant (MDR) Gram-negative bacterial infections, including CPE, beyond the urinary tract (23, 24). As with polymyxins, resistance to fosfomycin has also been reported in KPC-producing K. pneumoniae based upon susceptibility testing results (25, 26). Combination therapy is often employed to ensure adequate killing and suppress the emergence of resistance in difficult-to-treat infections.

Until novel agents become available, clinicians are limited to using existing antibiotics and combination therapy to combat KPC-producing K. pneumoniae. Alternative dosing strategies that limit the emergence of resistance need to be explored to optimize and preserve the use of these antibiotics. Previous *in vitro* studies have reported synergy between polymyxin and fosfomycin against carbapenem-resistant K. pneumoniae (27–30). Thus, the objectives of this study were to evaluate the pharmacodynamic (PD) activity of polymyxin B and fosfomycin alone and in combination against KPC-producing K. pneumoniae and to assess the rate and extent of emergence of resistance to different antibiotic regimens. We employed an *in vitro* hollow-fiber infection model (HFIM) to simulate clinically relevant dosing regimens over an extended duration.

## RESULTS

### Pharmacokinetic (PK) validation.

The polymyxin B free steady-state maximum concentration (*f*C_max,ss_) and free steady-state minimum concentration (*f*C_min,ss_) (mean ± standard deviation) were 3.63 ± 0.13 (*n* = 6) and 0.54 ± 0.02 mg/liter (*n* = 6) for the targets of 3.45 and 0.51 mg/liter, respectively. For a fosfomycin 1-h infusion, the *f*C_max,ss_ and *f*C_min,ss_ were 246 ± 18 (*n* = 8) and 111 ± 9 mg/liter (*n* = 8) for the targets of 250 and 105 mg/liter, respectively. For a fosfomycin 3-h infusion, *f*C_max,ss_ and *f*C_min,ss_ were 209 ± 18 (*n* = 8) and 125 ± 11 mg/liter (*n* = 8) for the targets of 212 and 126 mg/liter, respectively.

### Pharmacodynamic activity and emergence of resistance.

**FIG 1:**
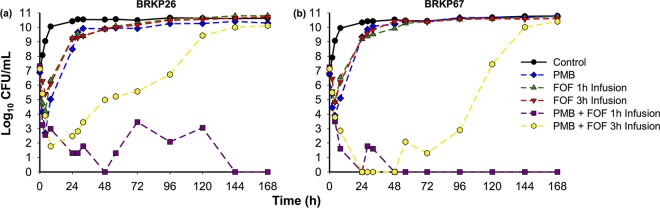
Time course of bacterial density in response to antibiotic regimens with polymyxin B (PMB) and fosfomycin (FOF) alone and in combination against an inoculum of ∼10^7^ CFU/ml of BRKP26 (polymyxin B MIC, 0.5 mg/liter; fosfomycin MIC, 32 mg/liter) and of BRKP67 (polymyxin B MIC, 8 mg/liter; fosfomycin MIC, 32 mg/liter) in a hollow-fiber infection model. PMB, polymyxin B 2.5-mg/kg loading dose as a 2-h infusion followed by 1.5 mg/kg q12h as a 1-h infusion; FOF 1-h infusion, fosfomycin at 6 g q6h as a 1-h infusion; FOF 3-h infusion, fosfomycin at 6 g q6h as a 3-h extended infusion; PMB + FOF 1-h infusion, combination of polymyxin B and a fosfomycin 1-h infusion; PMB + FOF 3-h infusion, combination of polymyxin B and a fosfomycin 3-h infusion.

**FIG 2:**
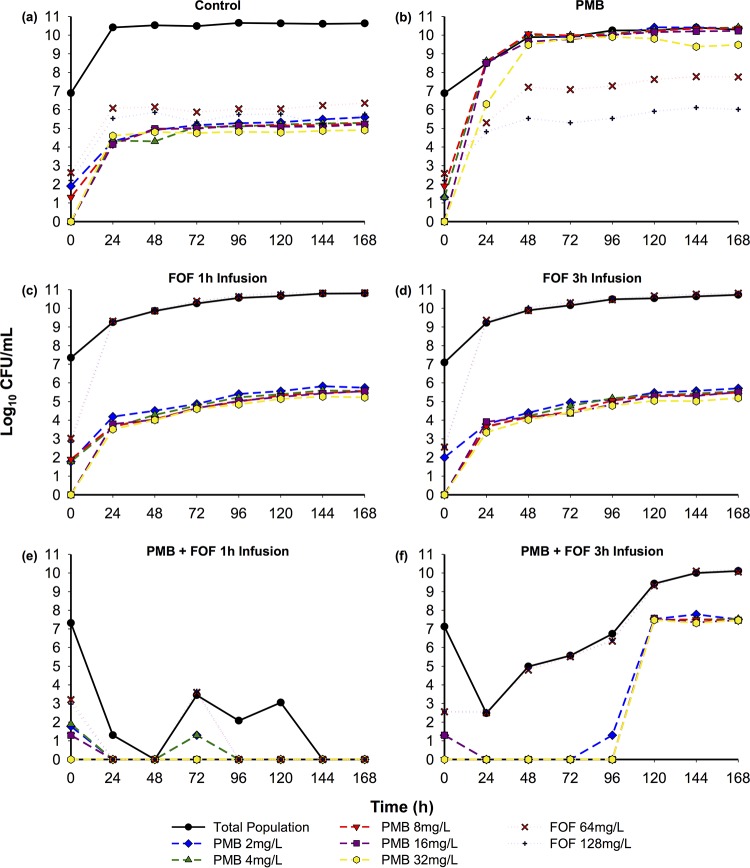
Time course of BRKP26 (polymyxin B MIC, 0.5 mg/liter; fosfomycin MIC, 32 mg/liter) subpopulations plated on polymyxin B (PMB)- or fosfomycin (FOF)-containing agar in response to antibiotic regimens in the hollow-fiber infection model. Dashed lines, subpopulations viable on polymyxin B; dotted lines, subpopulations viable on fosfomycin. The regimens, as indicated, were as follows: control, no treatment; PMB, polymyxin B 2.5-mg/kg loading dose as a 2-h infusion followed by 1.5 mg/kg q12h as a 1-h infusion; FOF 1-h infusion, fosfomycin at 6 g q6h as a 1-h infusion; FOF 3-h infusion, fosfomycin at 6 g q6h as a 3-h extended infusion; PMB + FOF 1-h infusion, combination of polymyxin B and a fosfomycin 1-h infusion; PMB + FOF 3-h infusion, combination of polymyxin B and a fosfomycin 3-h infusion.

**FIG 3:**
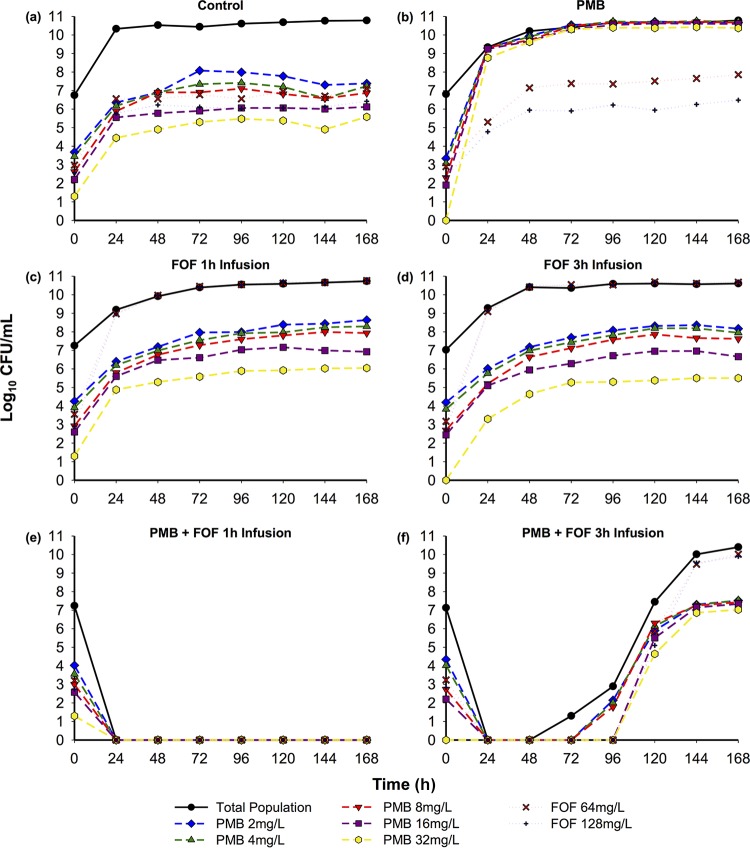
Time course of BRKP67 (polymyxin B MIC, 8 mg/liter; fosfomycin MIC, 32 mg/liter) subpopulations plated on polymyxin B (PMB)- or fosfomycin (FOF)-containing agar in response to antibiotic regimens in the hollow-fiber infection model. Dashed lines, subpopulations viable on polymyxin B; dotted lines, subpopulations viable on fosfomycin. The regimens, as indicated, were as follows: control, no treatment; PMB, polymyxin B 2.5-mg/kg loading dose as a 2-h infusion followed by 1.5 mg/kg q12h as a 1-h infusion; FOF 1-h infusion, fosfomycin at 6 g q6h as a 1-h infusion; FOF 3-h infusion, fosfomycin at 6 g q6h as a 3-h extended infusion; PMB + FOF 1-h infusion, combination of polymyxin B and a fosfomycin 1-h infusion; PMB + FOF 3-h infusion, combination of polymyxin B and a fosfomycin 3-h infusion.

The time course of bacterial density in response to antibiotic regimens evaluated against BRKP26 and BRKP67 and the related pharmacodynamic analysis are shown in Fig. 1 and Table 1. Population analysis profiles (PAPs) for BRKP26 and BRKP67 over 168 h are presented in Fig. 2 and 3. Pretreatment and posttreatment polymyxin B and fosfomycin MICs are listed in Table 2.

**TABLE 1:**
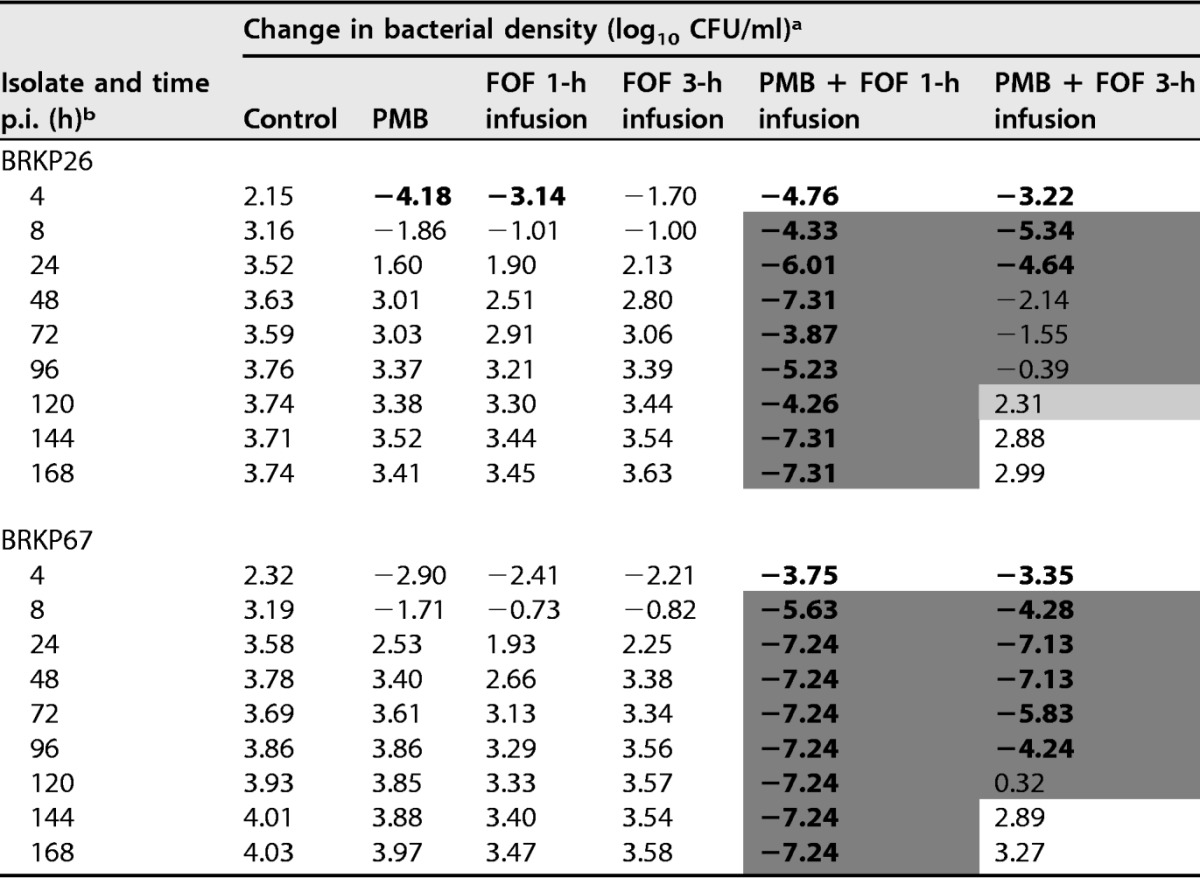
Change in bacterial density over time compared to that at baseline after treatment with antibiotic regimens in the hollow-fiber infection model against BRKP26 and BRKP67

^a^ Values represent changes in bacterial density relative to the level at baseline (0 h). Additivity (1 to <2 log_10_ CFU/ml-greater reduction) and synergy (≥2 log_10_ CFU/ml-greater reduction) with the combination compared to values of the most active single agent in the combination are highlighted in light gray and dark gray, respectively. Boldface type indicates bactericidal activity (≥3 log_10_ CFU/ml-reduction compared to the amount of the initial inoculum). The initial inoculum was ∼10^7^ CFU/ml. Regimens are as follows: control, no treatment; PMB, polymyxin B 2.5-mg/kg loading dose as a 2-h infusion followed by 1.5 mg/kg q12h as a 1-h infusion; FOF 1 h infusion, fosfomycin at 6 g q6h as a 1-h infusion; FOF 3-h infusion, fosfomycin at 6 g q6h as a 3-h extended infusion; PMB + FOF 1-h infusion, combination of polymyxin B and a fosfomycin 1-h infusion; PMB + FOF 3-h infusion, combination of polymyxin B and a fosfomycin 3-h infusion.

^b^ p.i., postinfection.

**TABLE 2:** Polymyxin B and fosfomycin susceptibility test results after treatment with antibiotic regimens in the hollow-fiber infection model for 168 h

Treatment regimen^*a*^	Antibiotic MIC for the indicated strain (mg/liter)			
	BRKP26		BRKP67	
	Polymyxin B	Fosfomycin	Polymyxin B	Fosfomycin
Baseline (0 h)	0.5	32	8	32
Control	0.5	32	8	32
PMB	128	64	128	32
FOF 1-h infusion	0.5	>256	8	>256
FOF 3-h infusion	<0.5	>256	16	>256
PMB + FOF 1-h infusion^*b*^	N/A	N/A	N/A	N/A
PMB + FOF 3-h infusion	64	>256	32	>256

a Regimens are as follows: control, no treatment; PMB, polymyxin B 2.5-mg/kg loading dose as a 2-h infusion followed by 1.5 mg/kg q12h as a 1-h infusion; FOF 1-h infusion, fosfomycin at 6 g q6h as a 1-h infusion; FOF 3 h infusion, fosfomycin at 6 g q6h as a 3-h extended infusion; PMB + FOF 1-h infusion, combination of polymyxin B and a fosfomycin 1-h infusion; PMB + FOF 3-h infusion, combination of polymyxin B and a fosfomycin 3-h infusion.

b No colonies were detected after treatment.

### Treatment regimens. (i) Control.

 The control arm (growth control) resulted in >3 logs of growth by 8 h for both isolates (Fig. 1). BRKP26 and BRKP67 were both polymyxin B- and fosfomycin-heteroresistant strains, with subpopulations growing on Mueller-Hinton II agar (MHA) plates containing antibiotic concentrations of >2× the MIC at baseline (0 h). Resistant subpopulations increased proportionally with increases in the total population (Fig. 2a and 3a).

### (ii) Polymyxin B monotherapy.

Polymyxin B monotherapy (2.5-mg/kg loading dose as a 2-h infusion followed by 1.5 mg/kg every 12 h [q12h] as a 1-h infusion) against BRKP26 demonstrated early bactericidal activity by 4 h, followed by regrowth similar to that of the control by 28 h (Fig. 1a). Against BRKP67, polymyxin B monotherapy resulted in a 2.9-log reduction by 4 h, followed by regrowth similar to that of the control by 28 h (Fig. 1b). Emergence of polymyxin B resistance in both strains was evident by 24 h; growth of the subpopulations on MHA containing 2 to 32 mg/liter of polymyxin B was similar to that of the total population (Fig. 2b and 3b). Growth of the fosfomycin-resistant subpopulations remained similar to that of the controls. Both BRKP26 and BRKP67 became highly resistant to polymyxin B, with MICs increased to 128 mg/liter after treatment, while the fosfomycin MIC did not significantly change (Table 2).

### (iii and iv) Fosfomycin monotherapy with 1-h and 3-h infusions.

Fosfomycin 1-h infusion monotherapy (6 g every 6 h [q6h] as a 1-h infusion) against BRKP26 demonstrated early bactericidal activity by 4 h, while a fosfomycin 3-h infusion (6 g q6h as a 3-h extended infusion) resulted in a 1.7-log reduction by 4 h. However, both regimens resulted in regrowth similar to that of the control by 28 h (Fig. 1a). Against BRKP67, both the fosfomycin 1-h infusion and fosfomycin 3-h infusion resulted in a >2-log reduction in growth by 4 h, followed by regrowth similar to that of the control by 28 h (Fig. 1b). Fosfomycin dosed as a 1-h or 3-h infusion against both strains resulted in the emergence of fosfomycin resistance by 24 h (Fig. 2c and d and 3c and d). Growth of the subpopulations on MHA containing 64 and 128 mg/liter of fosfomycin was similar to that of the total population, while growth of subpopulations on polymyxin B-containing plates remained similar to that of the control. The fosfomycin MIC of both strains increased to >256 mg/liter after treatment, while the polymyxin B MIC did not significantly change (Table 2).

### (v) Combination of polymyxin B and a fosfomycin 1-h infusion.

The combination of polymyxin B (ii) and a fosfomycin 1-h infusion (iii) was synergistic (Table 1). This regimen demonstrated sustained bactericidal activity beyond 4 h against both isolates; colony counts were undetectable beyond 144 h for BRKP26 and beyond 24 h for BRKP67 (Fig. 1). Polymyxin B plus a fosfomycin 1-h infusion suppressed the emergence of resistance. PAPs of BRKP26 revealed a fosfomycin-resistant subpopulation at 72 h; however, resistant subpopulations were undetectable beyond 96 h (Fig. 2e). PAPs of BRKP67 did not contain resistant subpopulations after treatment (Fig. 3e).

### (vi) Combination of polymyxin B and a fosfomycin 3-h infusion.

Polymyxin B (ii) plus a fosfomycin 3-h infusion (iv) was also synergistic and resulted in increased killing activity and delayed regrowth compared to growth with monotherapy (Table 1). The combination demonstrated bactericidal activity by 4 h against both isolates; however, unlike polymyxin B plus a fosfomycin 1-h infusion, the killing activity was not sustained. Polymyxin B plus a fosfomycin 3-h infusion was bactericidal up to 32 h against BRKP26 and up to 96 h against BRKP67; regrowth similar to that of the control was observed by 144 h (Fig. 1).

The combination of polymyxin B and a fosfomycin 3-h infusion resulted in the emergence of resistance to both antibiotics. PAPs of BRKP26 revealed polymyxin B-resistant subpopulations growing on MHA containing 2 to 32 mg/liter of polymyxin B beyond 120 h (Fig. 2f). Growth of the subpopulations on MHA containing 64 and 128 mg/liter of fosfomycin was similar to that of the total population beyond 24 h. PAPs of BRKP67 revealed amplification of polymyxin B resistance by 120 h; compared to control, a higher proportion of subpopulations grew on MHA containing 16 and 32 mg/liter of polymyxin B (Fig. 3a and f). Growth of the subpopulations on MHA containing 64 and 128 mg/liter of fosfomycin was similar to that of the total population beyond 144 h. After treatment, both strains became highly resistant to polymyxin B and fosfomycin, with MICs increasing to ≥32 and >256 mg/liter, respectively (Table 2).

## DISCUSSION

Multidrug-resistant (MDR) bacterial pathogens like CPE are at the center of difficult-to-treat infections and represent a major public health threat (1, 2). The pace of antibiotic discovery cannot keep up with the global and unabated spread of resistant pathogens (3, 6). The emergence of resistance to some of the newer antibiotic agents is concerning. Hence, the development of optimized regimens to better dose currently available agents against these MDR strains represents an alternative strategy to provide safe and effective treatment and suppress the emergence of resistance.

Here, we evaluated pharmacodynamic activity by simulating clinically relevant dosing regimens of polymyxin B and fosfomycin in monotherapy and in combination against KPC-producing K. pneumoniae using an *in vitro* hollow-fiber infection model. Furthermore, we assessed the rate and extent of the emergence of resistance to these simulated regimens using PAPs and susceptibility testing.

Monotherapy regimens with either polymyxin B (2.5-mg/kg loading dose as a 2-h infusion followed by 1.5 mg/kg q12h as a 1-h infusion) or fosfomycin (6 g q6h as a 1-h or 3-h infusion) were ineffective. Despite initial pharmacodynamic activity, there was rapid emergence of resistance with both of the monotherapies. No significant difference in results was seen between fosfomycin dosed as a 1-h infusion and fosfomycin dosed as a 3-h infusion. All regimens selected for resistance, with the more susceptible population being replaced by the resistant subpopulation by 24 h. Previous studies evaluating monotherapy with either polymyxin B or fosfomycin suggest the use of combination therapy to reduce the potential for developing resistance during treatment (11, 31, 32). In addition, monotherapy is reported to be associated with higher mortality rates than combination therapy in studies evaluating clinical outcomes of infections caused by KPC-producing K. pneumoniae (33, 34).

Polymyxin B in combination with fosfomycin was synergistic and resulted in increased pharmacodynamic activity with more extensive and sustained killing than that with the monotherapy regimens. This is consistent with previous *in vitro* time-kill and Etest studies reporting synergy between polymyxins and fosfomycin against KPC-producing K. pneumoniae (28, 29). In a small prospective observational study, Michalopoulos et al. evaluated combination therapy with intravenous fosfomycin (2 to 4 g q6h) for the treatment of hospital-acquired infections (bacteremia, ventilator-associated pneumonia, and urinary tract infection) caused by carbapenem-resistant K. pneumoniae in critically ill patients (24). Bacterial eradication and clinical cure or improvement were reported in the six patients treated with the combination.

Interestingly, complete bacterial eradication was observed with the combination of polymyxin B (2.5-mg/kg loading dose as a 2-h infusion followed by 1.5 mg/kg q12h as a 1-h infusion) and a fosfomycin 1-h infusion (6 g q6h as a 1-h infusion). However, emergence of populations resistant to both polymyxin B and fosfomycin appeared after treatment with the combination of polymyxin B (2.5-mg/kg loading dose as a 2-h infusion followed by 1.5 mg/kg q12h as a 1-h infusion) and a fosfomycin 3-h infusion (6 g q6h as an extended 3-h infusion). In a case report of KPC-producing K. pneumoniae infection, colistin (120,000 IU/kg/day in two divided doses) and fosfomycin (4 g q6h) initially relieved symptoms and controlled the bacteremia but was followed by clinical and microbiological relapse after 4 days of treatment due to the selection of resistant subpopulations (35). Unfortunately, the infusion duration was not reported. It is important to note that the PK/PD index, which best predicts fosfomycin efficacy against K. pneumoniae, remains unclear. Killing activity against Enterobacteriaceae has been reported to be dependent upon time, concentration, and the free steady-state area under the concentration-time curve (*f*AUC) (11, 36–38). Both fosfomycin dosing strategies simulated in the current study had the same overall exposures (*f*AUC over 24 h [*f*AUC_24_], 4,063 mg · h/liter) and achieved concentrations that exceeded the MIC for 100% of the dosing interval (percent time above the MIC, %*T*_MIC_) at steady state. The *f*C_max,ss_ values differed between regimens (250 mg/liter for a 1-h infusion and 212 mg/liter for a 3-h infusion), suggesting that fosfomycin in combination with polymyxin B may have concentration-dependent killing as the 1-h infusion was more effective. Of note, VanScoy et al. recently reported that the percentage of time that concentrations are above the MIC for the resistant subpopulation (%*T*_RIC_) is the PK/PD index most associated with efficacy (39). Target attainment of this index would be difficult to achieve if subpopulations have an MIC of >128 mg/liter; both fosfomycin dosing strategies in the present study would achieve a 0%*T*_RIC_ of ≥256 mg/liter. Further studies are needed to accurately elucidate a PK/PD index for K. pneumoniae.

Although the exact mechanism is unknown, the synergy seen here could be due to increased target site concentration of fosfomycin. Polymyxin B binds to lipopolysaccharide on the bacterial outer membrane, thereby disrupting its integrity. Consequently, this might enhance fosfomycin penetration into bacterial cells (22, 40). Additionally, polymyxin B may eradicate the bacterial subpopulations that are less susceptible to fosfomycin, and fosfomycin may eradicate the subpopulations that are less susceptible to polymyxin B (subpopulation synergy) (41), a mechanism that might be relevant here. Our PAP results revealed the emergence of a fosfomycin-resistant subpopulation at 72 h that was most likely eradicated after polymyxin B dosing since polymyxin B-resistant subpopulations were not present (Fig. 2e).

One limitation of our study was that two clinical isolates of KPC-producing K. pneumoniae were evaluated and may not be representative of all KPC-producing K. pneumoniae or CPE. Additionally, although the hollow-fiber infection model allows dosing regimens to be simulated over a clinical duration, this dynamic *in vitro* system does not consider the potential impact of the host immune response on bacterial eradication and antibiotic resistance. Moreover, strains developing resistance mechanisms may have lower biological fitness and pathogenicity. Further work using both *in vitro* and *in vivo* systems to evaluate an array of CPE isolates expressing a broad range of resistance-promoting genes is warranted.

In conclusion, here we demonstrate that the combination of polymyxin B and fosfomycin dosed as a 1-h infusion may be a viable treatment option against KPC-producing K. pneumoniae and may suppress the emergence of resistance. As treatment options remain extremely limited, studies to evaluate novel dosing strategies like front-loading (42) will enable the design of effective regimens against MDR Gram-negative pathogens. Such innovative approaches are necessary to optimize polymyxin B and fosfomycin combination therapy and preserve the utility of our last-line agents.

## MATERIALS AND METHODS

### Bacterial strains.

Two K. pneumoniae clinical isolates were utilized in this study (BRKP26 and BRKP67) obtained from patients admitted to the Instituto Dante Pazzanesse de Cardiologio, São Paulo, Brazil, during KPC outbreaks between June 2009 and June 2013. Both isolates are KPC-2 producers. Both isolates have wild-type *murA*, *glpT*, and *uhpT* sequences and are susceptible to fosfomycin (MIC, 32 mg/liter for both). BRKP26 has a wild-type *mgrB* sequence and is susceptible to polymyxin B (MIC, 0.5 mg/liter). BRKP67 has a mutation in *mgrB* (insertion of an IS*5*-like element, ISKpn13 [1,148 bp], between the +74 and +75 nucleotide position in the coding region) and is resistant to polymyxin B (MIC, 8 mg/liter). Polymyxin B and fosfomycin MICs were determined in triplicate by broth microdilution and agar dilution, respectively, as per guidelines of the Clinical and Laboratory Standards Institute (43). PCR was performed using primer sets for β-lactamase Ambler class A (GES and KPC), B (NDM, VIM, and IMP), and D (OXA-48 and -40) (44) and for *mgrB* (45), *murA*, *glpT*, and *uhpT* (46) as previously described (47).

### Antimicrobials and media.

Mueller-Hinton broth (Becton, Dickinson and Company, Sparks, MD) supplemented with calcium and magnesium (CAMHB; 25.0 mg/liter Ca^2+^, 12.5 mg/liter Mg^2+^) and Mueller-Hinton II agar (MHA; Becton, Dickinson and Company, Sparks, MD) were used for susceptibility testing and all *in vitro* models. CAMHB and MHA were supplemented with 25 mg/liter glucose-6-phosphate (lot number 342377; Acros Organics, Bridgewater, NJ) for fosfomycin-containing experiments. Stock solutions of polymyxin B (lot number WXBB5309V; Sigma-Aldrich, St. Louis, MO) and fosfomycin (ZTI-01; lot number K001; Zavante Therapeutics, Inc., San Diego, CA) were freshly prepared in sterile water prior to each experiment. All drug solutions were filter sterilized using a 0.22-μm-pore-size filter (Fisher Scientific, Pittsburgh, PA).

### Hollow-fiber infection model.

A hollow-fiber infection model (HFIM) was used as previously described (42, 48) to evaluate polymyxin B and fosfomycin monotherapy and combination therapy regimens against BRKP26 and BRKP67 over 168 h. Cellulosic cartridges (FiberCell Systems, Frederick, MD) were utilized in all experiments. Briefly, fresh bacterial colonies from overnight growth were added to prewarmed CAMHB (37°C) to achieve an initial inoculum of ∼10^7^ CFU/ml. This logarithmic-phase broth culture was inoculated into the extracapillary space of the cellulosic cartridge. Peristaltic pumps (Masterflex L/S; Cole-Parmer, Vernon Hills, IL) were used to provide continuous CAMHB flow through the central compartment to simulate a half-life (*t*_1/2_) of 4 h (49–51). Multichannel syringe pumps (New Era Pump Systems, Farmingdale, NY) were used for antibiotic dosing into the central compartment. A Duet Pump (FiberCell Systems) provided continuous circulation between the central and peripheral compartments to ensure instantaneous drug distribution and homogeneous mixing. The HFIM was maintained at 37°C, and drug solutions were prepared daily and stored at 4°C immediately prior to dosing. The experimental design consisted of the following simulated regimens: (i) control, no treatment; (ii) polymyxin B monotherapy, consisting of a 2.5-mg/kg loading dose as a 2-h infusion followed by 1.5 mg/kg q12h as a 1-h infusion (free steady-state area under the concentration-time curve over 24 h [*f*AUC_24_], 38.0 mg · h/liter; free steady-state maximum concentration [*f*C_max,ss_], 3.45 mg/liter; free steady-state minimum concentration [*f*C_min,ss_], 0.51 mg/liter); (iii) fosfomycin as a 1-h infusion monotherapy, consisting of 6 g q6h as a 1-h infusion (*f*AUC_24_, 4,063 mg · h/liter; *f*C_max,ss_, 250 mg/liter; *f*C_min,ss_, 105 mg/liter); (iv) fosfomycin as a 3-h infusion monotherapy, consisting of 6 g q6h as a 3-h extended infusion (*f*AUC_24_, 4,063 mg · h/liter; *f*C_max,ss_, 212 mg/liter; *f*C_min,ss_, 126 mg/liter); (v) a combination of polymyxin B and a fosfomycin 1-h infusion; (vi) a combination of polymyxin B and a fosfomycin 3-h infusion.

The simulated polymyxin B regimen was based on the PK observed in critically ill patients (52). Simulated fosfomycin regimens were based on published clinical PK studies with a total daily dose of 24 g (49–51). Both 1-h and 3-h infusion dosing strategies were evaluated as fosfomycin administration as a short-term infusion may result in a different response from that with the extended infusions. Short-term infusions yield higher maximum concentrations while extended infusions maximize trough concentrations to provide a higher percentage of time above a target concentration at steady state.

Samples were obtained at 0, 2, 4, 8, 24, 28, 32, 48, 56, 72, 96, 120, 144, and 168 h for bacterial quantification. After serial dilution with sterile saline, samples were plated (50 μl) on MHA plates using a Whitley Automated Spiral Plater II (Don Whitley Scientific, Shipley, UK). Colony counts (log_10_ CFU per milliliter) were quantified using a ProtoCOL HR automated bacterial colony counter (Synbiosis, Frederick, MD) after 24 h of incubation at 37°C; the limit of quantification was 2 log_10_ CFU/ml. Two additional sets of samples (500 μl) were stored at −80°C until pharmacokinetic validation.

### Emergence of resistance.

To assess for the emergence of resistance, population analysis profiles (PAPs) were determined by plating samples collected at 0 (baseline), 24, 48, 72, 96, 120, 144, and 168 h on polymyxin B-containing MHA (2, 4, 8, 16, and 32 mg/liter) and on fosfomycin-containing MHA (64 and 128 mg/liter) for all the regimens evaluated. In addition to PAPs, MIC determination (43) was performed with HFIM samples collected at baseline and 168 h. MIC testing was performed in triplicate, and modal values are reported. A greater than 2-fold change in MIC after treatment compared to baseline was considered a significant difference.

### Pharmacokinetic validation.

Polymyxin B1 and B2 concentrations were quantified using a validated liquid chromatography tandem mass spectrometry (LC-MS/MS) assay (31). Analysis of independently prepared quality control samples indicated good reproducibility (coefficients of variation of ≤7.89%) and accuracy (measured concentrations of ≤10.5% from target concentrations). The limit of quantification was 0.025 mg/liter. Fosfomycin concentrations were quantified using a previously described biological assay (48). The fosfomycin standard curve was logarithmic over concentrations ranging from 50 to 800 mg/liter. The limit of quantification was 50 mg/liter.

### Pharmacodynamic analysis.

Pharmacodynamic effect was quantified as the change in log_10_ CFU/milliliter at time *t* ([CFU_*t*_] 4, 8, 24, 48, 72, 96, 120, 144, and 168 h) compared to the level at baseline (0 h [CFU_0_]) as follows: log change = log_10_(CFU_*t*_) − log_10_(CFU_0_). Bactericidal activity was defined as a ≥3-log_10_ CFU/ml reduction compared to the initial inoculum. Additivity and synergy were defined as reductions greater than 1- to <2-log_10_ CFU/ml and ≥2-log_10_ CFU/ml by the combination compared to the most active single agent in the combination, respectively.
